# Type I interferon signaling mediates *Mycobacterium tuberculosis*–induced macrophage death

**DOI:** 10.1084/jem.20200887

**Published:** 2020-10-30

**Authors:** Li Zhang, Xiuju Jiang, Daniel Pfau, Yan Ling, Carl F. Nathan

**Affiliations:** Department of Microbiology and Immunology, Weill Cornell Medicine, New York, NY

## Abstract

Macrophages help defend the host against *Mycobacterium tuberculosis* (Mtb), the major cause of tuberculosis (TB). Once phagocytized, Mtb resists killing by macrophages, replicates inside them, and leads to their death, releasing Mtb that can infect other cells. We found that the death of Mtb-infected mouse macrophages in vitro does not appear to proceed by a currently known pathway. Through genome-wide CRISPR-Cas9 screening, we identified a critical role for autocrine or paracrine signaling by macrophage-derived type I IFNs in the death of Mtb-infected macrophages in vitro, and blockade of type I IFN signaling augmented the effect of rifampin, a first-line TB drug, in Mtb-infected mice. Further definition of the pathway of type I IFN–mediated macrophage death may allow for host-directed therapy of TB that is more selective than systemic blockade of type I IFN signaling.

## Teaser

*Mycobacterium tuberculosis* (Mtb) infects macrophages and eventually leads to their death. Zhang et al. find that type I IFN signaling contributes to the death of Mtb-infected macrophages through an apparently novel mechanism.

## Introduction

*Mycobacterium tuberculosis* (Mtb), the causative agent of tuberculosis (TB), appears to have coevolved with humans over at least 70,000 yr and is estimated to latently infect about a quarter of the global population. Mtb causes clinically active TB in ∼10 million people each year and kills an average of three people a minute (World Health Organization, 2019). Along with recent advances to modernize an often toxic chemotherapeutic regimen that is losing ground to antimicrobial resistance (Conradie et al., 2020) and to improve on the existing vaccine (Darrah et al., 2020), attention has also been given to the possibility of adjunctive host-directed therapy (Frank et al., 2019; Tiberi et al., 2018). The goals of host-directed therapy would be to help shorten anti-mycobacterial regimens that now last 6–28 mo or more and to limit immunopathology. Inflammation at sites of TB disease continues even after clinical cure (Malherbe et al., 2016). Immunopathology compromises lung function and may contribute to the increased risk of developing TB again in people who have been cured of it (Verver et al., 2005). Better understanding of the host–pathogen interaction would help in devising host-directed therapies to shorten antimicrobial treatment and reduce tissue damage.

Type I IFNs are a large family of IFNs that are broadly implicated in host immune response to viral and bacterial infections. Type I IFN signaling is crucial mainly for host defense against viruses but also against some bacteria, such as group B streptococci, pneumococci, and *Escherichia coli* (Mancuso et al., 2007). However, type I IFNs promote infection by other bacteria, including Mtb (Manca et al., 2005; Mayer-Barber et al., 2014; Robinson et al., 2012; Stanley et al., 2007; Teles et al., 2013). A blood cell transcriptome indicative of response to type I IFNs is a signature of active TB (Berry et al., 2010). The signature can be evident 18 mo before TB diagnosis (Scriba et al., 2017), consistent with the possibility that type I IFN signaling may foster conversion of the disease from its latent to its active form. A genetic mutation in IFN-α and -β receptor subunit 1 (IFNAR1) that reduced binding of type I IFNs and was associated with increased susceptibility to viral hepatitis, suggesting its functional significance, was also associated with decreased susceptibility to TB (Zhang et al., 2018). Conversely, active TB has developed during administration of type I IFN to patients with hepatitis (Babudieri et al., 2012; Belkahla et al., 2010; Telesca et al., 2007) and multiple sclerosis (Sirbu et al., 2020).

Macrophages produce type I IFNs in vitro when infected with Mtb and in vivo in Mtb-infected hosts (Cheng and Schorey, 2018; Collins et al., 2015; Dey et al., 2015; Manca et al., 2005; Stanley et al., 2007; Wassermann et al., 2015; Watson et al., 2015). In mouse models of TB, host genetic background affects the impact of type I IFN signaling. Necrotic pulmonary lesions are prominent in mice bearing a hypomorphic allele of *Sp140* in the “*Super susceptibility to tuberculosis 1* (*Sst1*)” locus (Kramnik et al., 2000) that impairs the ability of *Sp140* to suppress the type I IFN response during intracellular bacterial infections (Ji et al., 2020 *Preprint*). In C57BL/6 (B6) congenic mice carrying the “susceptible” allele of the *Sst1* locus, heightened tissue damage has been related to increased production of IL-1 receptor antagonist (Ji et al., 2019). However, type I IFNs induce the expression of several hundred genes, and the mechanisms by which they impair host control of Mtb are likely to be multifactorial as well as related to host genetic background.

Mtb establishes chronic infection chiefly by parasitizing macrophages. After macrophages phagocytize the bacterium, Mtb inhibits maturation of the phagosome into acidified phagolysosomes (Sturgill-Koszycki et al., 1994), promoting the pathogen’s survival and replication. If no Mtb-active antibiotics are used during the preparation and infection of macrophages in vitro, virulent Mtb kills the macrophages. If the investigator uses a very low multiplicity of infection (MOI; e.g., 0.1) and an optimal combination of oxygen tension, plasma concentration, and macrophage-activating cytokines, death of macrophage cultures is only postponed by a few weeks (Vogt and Nathan, 2011). In the host as well, macrophages die and release bacteria that can infect other cells. Thus, Mtb-induced cell death is a key factor in TB pathogenesis (Behar et al., 2010; Behar et al., 2011) and possibly a point for therapeutic intervention. Death of macrophages infected with virulent Mtb reportedly involves phagosomal rupture, mitochondrial dysfunction, ROS generation, and breaks in the plasma membrane (Lee et al., 2011; Zhao et al., 2017) or mycobacterial degradation of the macrophages’ nicotinamide adenine dinucleotide (Pajuelo et al., 2018). However, there has been no identification of an upstream point of control at which Mtb-infected macrophages instruct themselves to undertake their own death.

Along with a lack of certainty about its upstream control points and downstream mechanisms, it is uncertain how the death of Mtb-infected macrophages compares with forms of cell death characterized in other settings (Galluzzi et al., 2018). Apoptosis, the first clearly delineated mode of programmed cell death, plays an important role in development as well as immune defense against pathogens. Apoptosis was reported in macrophages infected with attenuated Mtb as well as virulent Mtb and was dependent on the autocrine or paracrine action of TNFα (Keane et al., 1997). Later studies concluded that virulent Mtb suppresses macrophage apoptosis (Behar et al., 2010; Keane et al., 2000). The role of necroptosis, a form of programmed necrotic cell death, remains controversial in TB (Pajuelo et al., 2018; Stutz et al., 2018a; Stutz et al., 2018b; Zhao et al., 2017). TNF-related cell necrosis was reported in *Mycobacterium marinum*–infected zebrafish in vivo and *M. marinum*–infected macrophage cell lines in vitro without conforming to the current model of necroptosis (Roca et al., 2019), but that study did not involve Mtb or primary mammalian macrophages. Pyroptosis, a form of programmed necrotic cell death mediated by gasdermin proteins (Kayagaki et al., 2015; Shi et al., 2017; Shi et al., 2015; Wang et al., 2017), usually involves inflammasome activation and the maturation and release of pro-inflammatory cytokines, including IL-1β. Infection of macrophages with Mtb was reported to activate the NLRP3 and/or AIM2 inflammasomes, for both of which the adaptor ASC is essential (Dorhoi et al., 2012; Koo et al., 2008; Kurenuma et al., 2009; Wassermann et al., 2015). However, *Nlrp3*^−/−^ mice control TB similarly to WT mice (Dorhoi et al., 2012). Mice deficient in IL-1 receptor signaling are far more susceptible to TB than *Casp1*^−/−^ (actually *Casp1*^−/−^ *Casp11*^−/−^; Kayagaki et al., 2011; Kuida et al., 1995) and *Asc*^−/−^ mice are, suggesting a role for caspase-1– and caspase-11–independent IL-1β production and arguing against the importance of pyroptosis in TB (Dorhoi et al., 2012; Mayer-Barber et al., 2010). Recently, it was proposed that ferroptosis characterizes the death of Mtb-infected macrophages (Amaral et al., 2019).

Here, we report an analysis of macrophage cell death upon Mtb infection that suggests that apoptosis, pyroptosis, necroptosis, parthanatos, ferroptosis, and autophagy-dependent cell death are not the dominant cell death modes under the conditions studied. Our genome-wide screen and subsequent genetic and immunological studies show that autocrine or paracrine type I IFN signaling plays a controlling role in fostering the death of Mtb-infected macrophages.

Our findings reveal the central role of type I IFN signaling in Mtb-induced macrophage cell death, reinforce the idea of blocking type I IFN signaling as a host-directed therapy against TB, and raise the possibility that blocking a specific downstream step in type I IFN signaling could protect macrophages from Mtb.

## Results and discussion

### Mtb-induced macrophage death is likely to involve a novel mechanism

**Figure 1.**
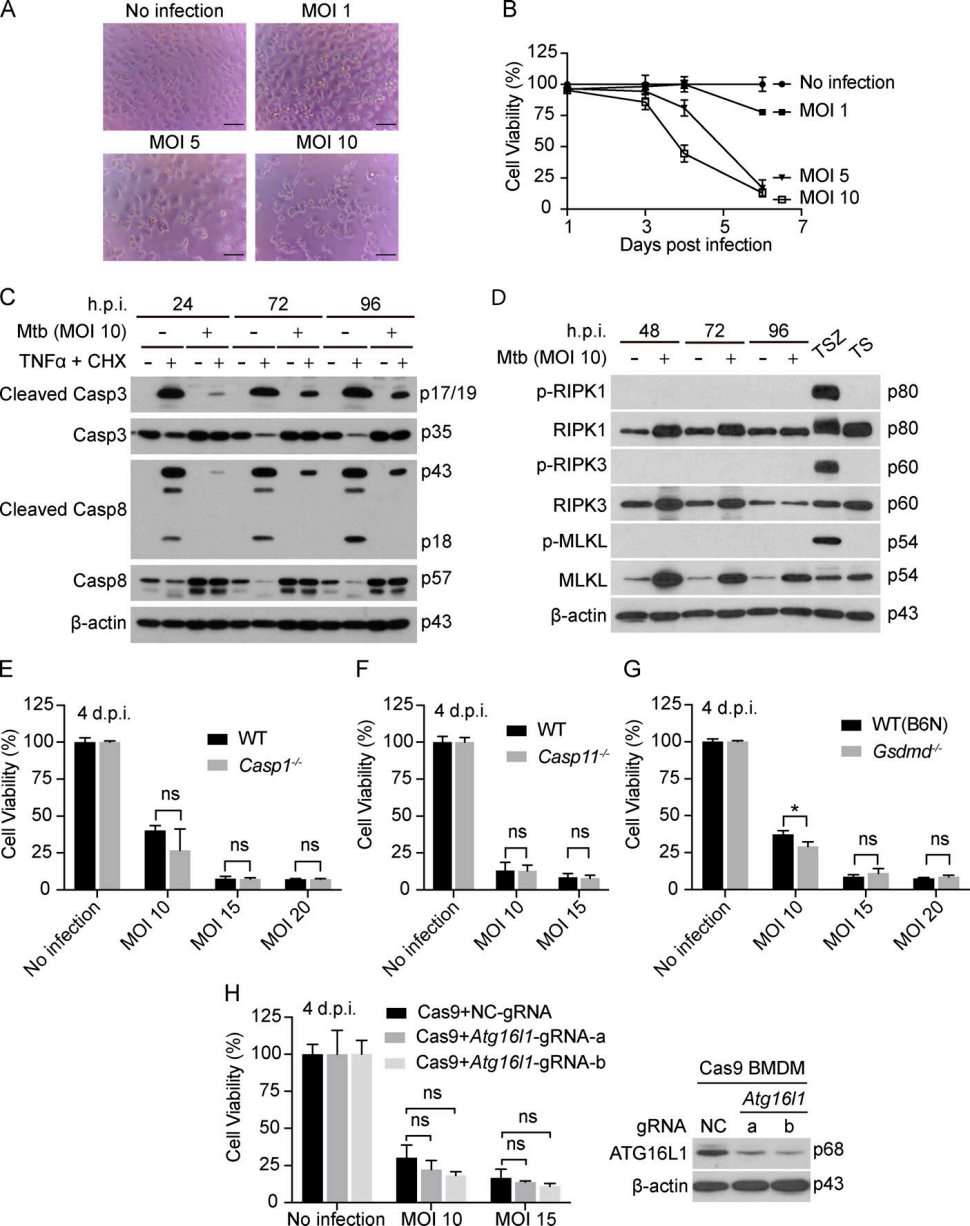
**Mtb induces death of BMDMs without hallmarks of apoptosis, necroptosis, pyroptosis or autophagic cell death.** **(A and B)** BMDM death after Mtb infection. Cells were infected with Mtb at the indicated MOI. **(A)** Photomicrographs were taken at 4 d postinfection (d.p.i.). Scale bar, 50 µm. **(B)** ATP levels were measured at the indicated times as an index of cell viability. **(C and D)** Effects of Mtb infection on BMDM apoptosis and necroptosis. Cells were infected or not with Mtb at an MOI of 10. At the indicated hours post infection (h.p.i.) and at the corresponding time for uninfected cells, some of the cells were additionally treated with TNFα (10 ng/ml) and CHX (10 µg/ml) for 2.5 h (C) or with the combination of TNFα (T; 10 ng/ml) and Smac-mimetic birinapant (S; 10 µM) with or without z-VAD-FMK (Z; 20 µM; D) before SDS-PAGE and immunoblots of cell lysates with the indicated antibodies. **(E–H)** Effects of deficiency of caspase-1, caspase-11, *Gsdmd*, or *Atg16l1* (see Materials and methods for details; a and b refer to two different gRNAs targeting *Atg16l1*) on Mtb-induced macrophage cell death. BMDMs were infected at the indicated MOI, and viability was assayed on 4 d.p.i. by ATP assay. Molecular weight of target proteins in the form of p followed by the indicated molecular mass in kD is labeled on the right of Western blots (C, D, and H). Data are means ± SD of three technical replicates (B and E–H). *, P < 0.05 (two-tailed unpaired Student’s *t* test). Data are representative of three (C) and two (D and E–H) independent experiments. NC, negative controls; ns, not significant.

Mtb infection of macrophages, including mouse bone marrow–derived macrophages (BMDMs), leads to death of the macrophages (Fig. 1, A and B), but the mechanism remains elusive. We first asked whether the cell death fell into any of the known death categories implicated in earlier studies.

**Figure S1.**
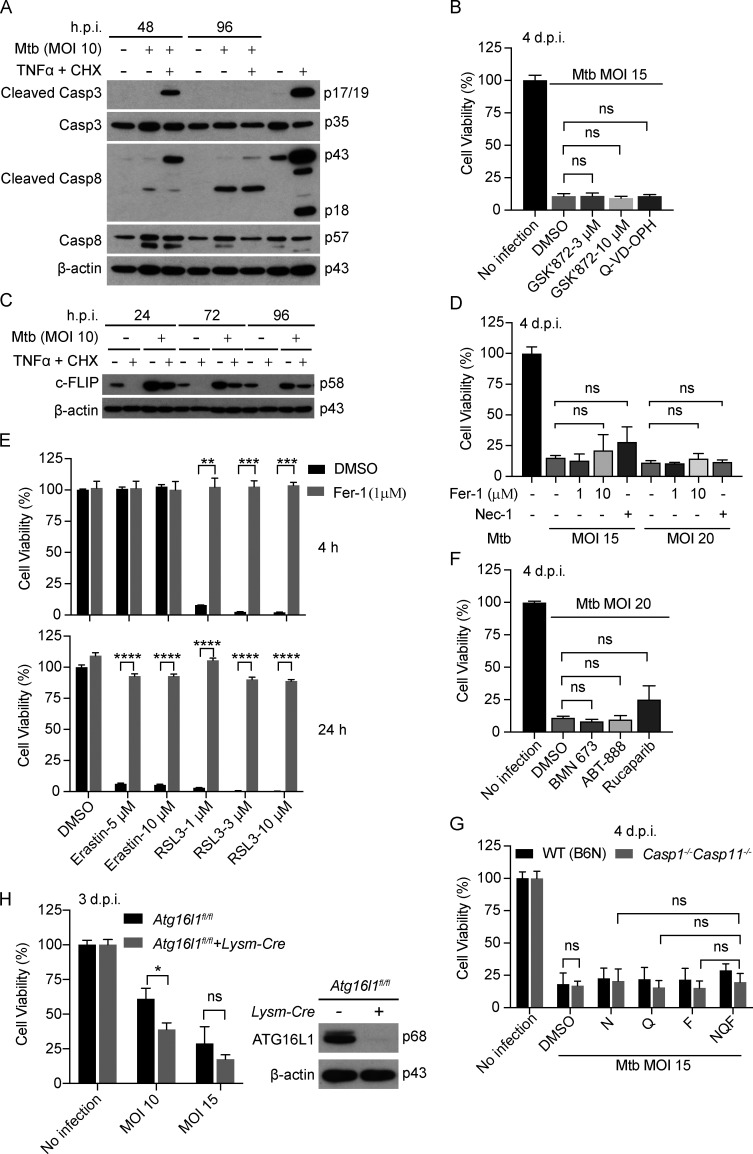
**Mtb-infected macrophages do not show markers of apoptosis, and their death is not likely to involve ferroptosis, necroptosis, parthanatos, or autophagic cell death.** Related to Fig. 1. **(A)** Effects of Mtb infection on macrophage apoptosis. WT BMDMs were infected or not with Mtb at an MOI of 10. At the indicated hours postinfection (h.p.i.) and at the corresponding time for uninfected cells, some of the cells were additionally treated with TNFα (10 ng/ml) and CHX (10 µg/ml) for 2.5 h before SDS-PAGE and immunoblots of cell lysates with the indicated antibodies. **(B)** Effects of GSK′872 and Q-VD-OPH on Mtb-induced macrophage death. WT BMDMs were pretreated with compounds at half of the indicated concentration for 2 h before infection of Mtb and then indicated concentration (GSK′872 as shown in figure and 20 μM for Q-VD-OPH) after washing Mtb away, and compound treatment continued throughout the experiment. Cell viability was measured by ATP assay on day 4. **(C)** Effects of Mtb infection on c-FLIP expression. WT BMDMs were infected with Mtb at an MOI of 10 for the indicated time and treated with TNFα (10 ng/ml) and CHX (10 µg/ml) 2.5 h before harvest. Shown are immunoblots of cell lysates. **(D)** Effects of Fer-1 and Nec-1 on Mtb-induced macrophage death. WT BMDMs were treated, infected, and assayed as in B. Nec-1 was used at 20 µM and Fer-1 as indicted in the figure. **(E)** Effects of Fer-1 on ferroptosis induced by Erastin and RSL3. HT-1080 cells were treated with compound combinations as indicated, and cell viability was measured by ATP assay on indicated time after treatment. **(F)** Effects of PARP inhibitors on Mtb-induced macrophage death. WT BMDMs were treated, infected, and assayed as in B. Compounds were all used at 10 µM. **(G)** Effects of the blockade of pyroptosis, ferroptosis, necroptosis, and caspase functions on Mtb-induced macrophage death. WT or *Casp1*^−/−^ *Casp11*^−/−^ BMDMs were treated, infected, and assayed as in B. N, Nec-1, 20 µM; Q, Q-VD-OPH, 20 µM; F, Fer-1, 10 µM. **(H)** Effects of ATG16L1 deficiency on Mtb-induced macrophage death. BMDMs were infected with Mtb at indicated MOIs and assayed on day 3 by ATP assay. Right panel is the Western blot of cell lysate. Molecular weight of target proteins in the form of p followed by the indicated molecular mass in kD is labeled on the right of Western blots (A, C, and H). Data are means ± SD of three technical replicates (B and D–H) and are representative of two independent experiments, except that H was done once due to COVID-19–limited resources. *, P < 0.05; **, P < 0.01; ***, P < 0.001; ****, P < 0.0001 (two-tailed unpaired Student’s *t* test). d.p.i., days postinfection; ns, not significant.

TNFα is induced by mycobacterial infection in vitro and in vivo and plays a dual role, either protecting or harming the host (Bean et al., 1999; Botha and Ryffel, 2003; Tobin et al., 2010). TNFα plus cycloheximide (CHX) treatment in vitro triggered apoptosis in various types of cells (Wang et al., 2008), as marked by cleavage of caspase-8 and caspase-3, important contributors to apoptosis. In contrast, Mtb-infected BMDMs showed no signs of apoptotic activation (Fig. 1 C and Fig. S1 A), and death was not blocked by the pan-caspase inhibitor Q-VD-OPH (Fig. S1 B), consistent with the lack of membrane blebbing (Fig. 1 A). Instead, Mtb infection inhibited the cleavage of caspase-8 and caspase-3 triggered by TNFα + CHX (Fig. 1 C and Fig. S1 A). Inhibition of apoptosis correlated with a marked increase in the anti-apoptotic protein cellular FLICE (FADD-like IL-1β-converting enzyme)–inhibitory protein (c-FLIP) after Mtb infection (Fig. S1 C).

When TNFα signaling is combined with inhibition of the NF-κB pathway and inhibition of caspases, it can lead to necroptosis, as marked by the mechanistically relevant phosphorylation of receptor-interacting protein kinase 1 (RIPK1), RIPK3, and mixed-lineage kinase domain–like pseudokinase (MLKL; Holler et al., 2000; He et al., 2009; Sun et al., 2012; Wang et al., 2014). However, Mtb infection did not activate necroptosis, even though MLKL, the executioner of necroptosis, was greatly up-regulated by Mtb infection (Fig. 1 D). Consistent with this, necrostatin-1 (Nec-1), a well-known necroptosis inhibitor that targets RIPK1 (Degterev et al., 2008), and GSK′872 (Kaiser et al., 2013), a RIPK3 inhibitor, both failed to inhibit the death of Mtb-infected BMDMs (Fig. S1, B and D).

Recently, ferroptosis was reported to play an important role in Mtb-induced macrophage cell death (Amaral et al., 2019). However, we were unable to confirm protection of Mtb-infected macrophages by exposure to the ferroptosis inhibitor ferrostatin-1 (Fer-1; Fig. S1 D), even though the same stock of Fer-1 inhibited classic ferroptosis induced by Erastin and RSL3 (Fig. S1 E).

Inhibitors of poly(ADP-ribose) polymerase 1, such as BMN 673, ABT-888, and rucaparib, are often used to inhibit parthanatos, a type of regulated cell death related to hyperactivation of poly(ADP-ribose) polymerase 1, a component of DNA damage response machinery (Galluzzi et al., 2018). These three compounds failed to inhibit Mtb-induced macrophage cell death (Fig. S1 F), suggesting that parthanatos is unlikely to account for the death of the Mtb-infected macrophages studied here.

Autophagy can also contribute to cell demise in certain settings (Galluzzi et al., 2018). However, when we used the CRIPSR-Cas9 technique in WT BMDMs to knock down autophagy-related 16–like 1 (ATG16L1), an important player in autophagy, Mtb-induced cell death was undiminished (Fig. 1 H). Likewise, BMDMs from mice with a conditional knockout of ATG16L1 in macrophages were not protected from Mtb-induced cell death (Fig. S1 H), although the latter experiment could only be performed once because of a reduction in mouse inventory during the COVID-19 pandemic.

Pyroptosis requires the pore-forming activity of gasdermin proteins in response to cleavage by the inflammatory caspase-1 and -11/4/5 or by noninflammatory caspases (Kayagaki et al., 2015; Shi et al., 2017; Shi et al., 2015; Wang et al., 2017). Infection of macrophages with Mtb leads to activation of the NLRP3 and/or AIM2 inflammasomes (Dorhoi et al., 2012; Koo et al., 2008; Kurenuma et al., 2009; Wassermann et al., 2015), but caspase-1 and caspase-11 (the latter mentioned because of a spontaneous loss-of-function mutation in the 129S2/SvPas [129S2] strain used as donors of embryonic stem cells in the generation of *Casp1*^−/−^ mice) were found not to be involved in Mtb-induced macrophage cell death (Lee et al., 2011). We excluded a role for pyroptosis by observing that the Mtb-induced death of macrophages was unimpeded by individual or combined genetic deficiency of *Casp1* and *Casp11* or deficiency of gasdermin D (*Gsdmd*; Fig. 1, E–G; and Fig. S1 G).

Moreover, we considered that blocking one type of cell death might cause Mtb-infected cells to switch to another cell death pathway. However, the pan-caspase inhibitor Q-VD-OPH did not prevent Mtb-induced macrophage death, nor did a combination of Q-VD-OPH with inhibitors of necroptosis and ferroptosis when all three agents were applied together to BMDMs that were genetically pyroptosis proficient or pyroptosis deficient (*Casp1*^−/−^ *Casp11*^−/−^; Fig. S1 G). Based on these findings, Mtb-induced macrophage death under these conditions is likely to involve a novel mechanism.

### Genome-wide CRISPR-Cas9 screen identifies type I IFN signaling as important for the death of Mtb-infected RAW264.7 cells

**Figure 2.**
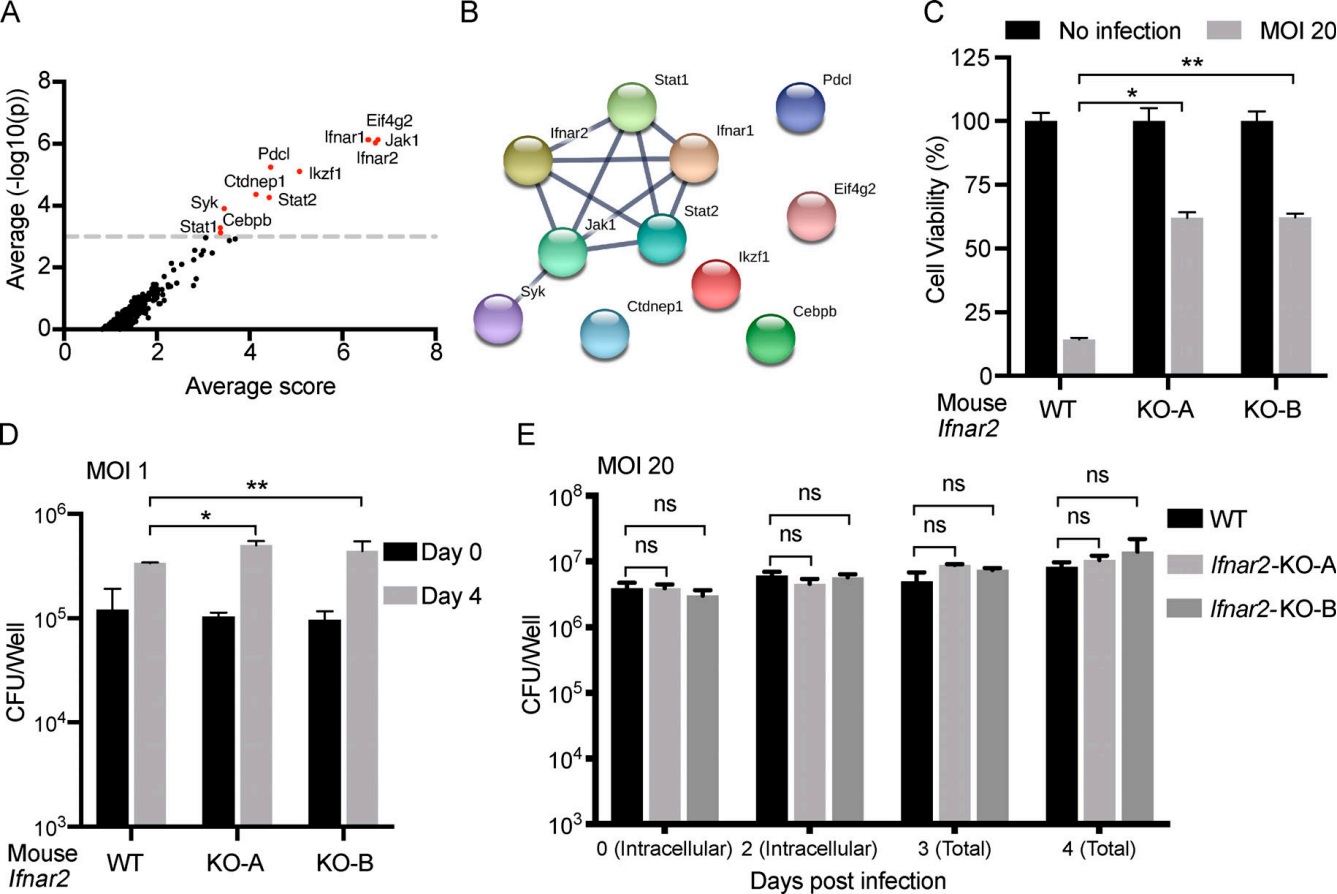
**Genome-wide CRISPR-Cas9 screen identifies type I IFN signaling as important for death of Mtb-infected RAW264.7 cells.** **(A)** Candidate gene hits from a genome-wide CRISPR-Cas9 screen of Mtb-induced RAW264.7 cell death. Shown are genes whose loss of function is beneficial for cell survival (positive screen) from three replicate experiments, where the x axis shows the calculated score using negative binomial distribution and the y axis represents the statistical analysis. Genes with average −log10(p) above 3 are highlighted in red. **(B)** Protein cluster analysis of gene hits identified in CRISPR-Cas9 screen. Gene hits identified from A were analyzed by the STRING database, and the interaction score cutoff was set as high confidence (0.7). **(C–E)** Results for two *Ifnar2*^−/−^ RAW264.7 clones (KO-A and KO-B) independently generated by CRISPR-Cas9–mediated targeting. **(C)** Cells were infected at an MOI of 20, and viability was assessed on day 3 from the adherent cell content of lactate dehydrogenase compared with that of uninfected control cultures. **(D and E)** Effects of *Ifnar2* deficiency on intracellular replication of Mtb. Cells were infected at an MOI of 1 (D) or 20 (E), and CFU of Mtb was determined by plating dilutions of lysates on 7H11 agar on the indicated days. Total represents the sum of intracellular and extracellular CFU. Data are means ± SD of two (C and D) or three (E) technical replicates. *, P < 0.05; **, P < 0.01 (two-tailed unpaired Student’s *t* test). Data are representative of two independent experiments. ns, not significant.

**Figure S2.**
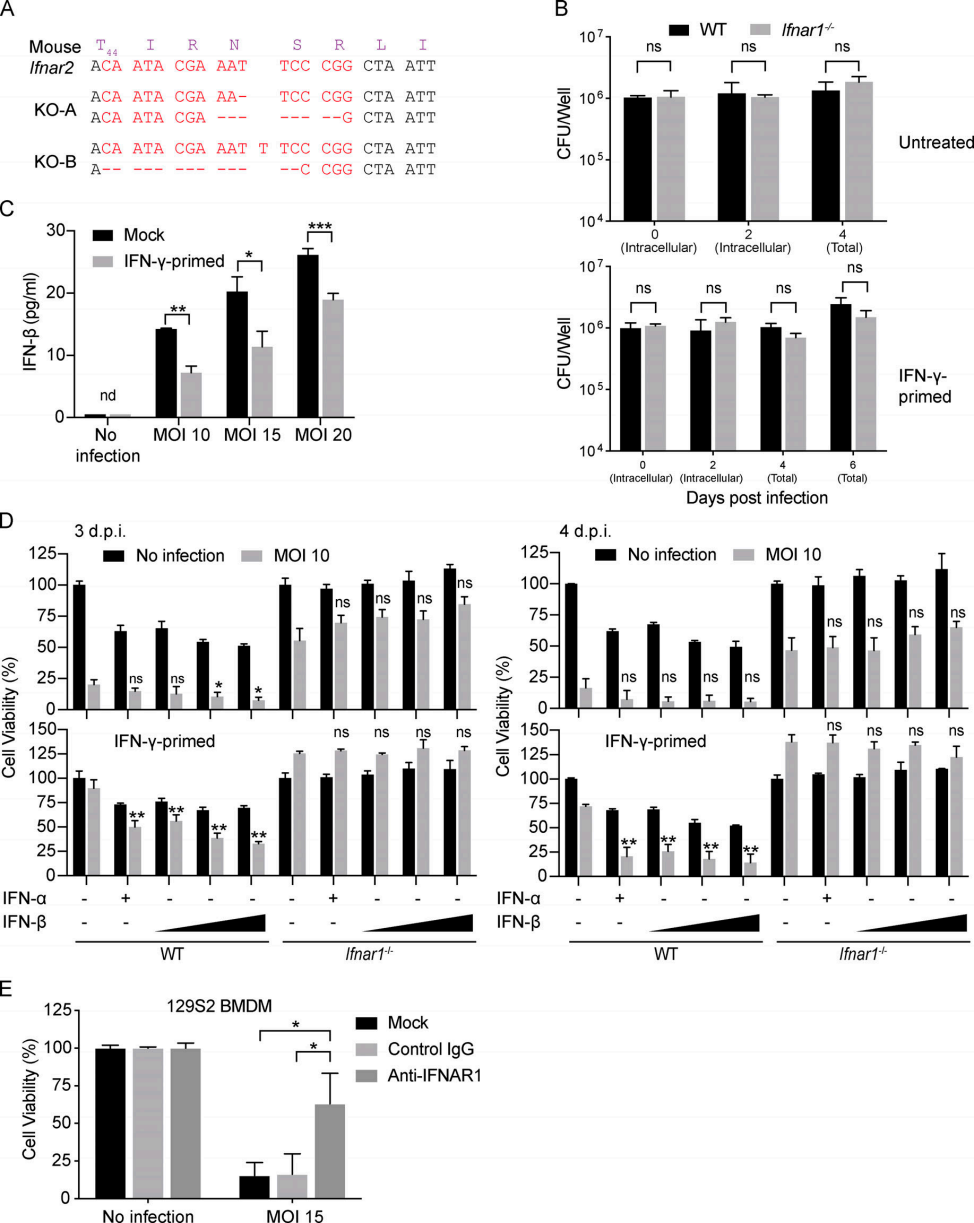
**Type I IFN signaling is important for Mtb-induced macrophage cell death.** Related to Figs. 2 and 3. **(A)** Genotypes of two individual RAW264.7 *Ifnar2*^−/−^ clones. **(B)** Effects of *Ifnar1* deficiency on replication of Mtb in BMDMs at an MOI of 20. IFN-γ priming at 10 ng/ml overnight. Total represents the sum of intracellular and extracellular CFU. **(C)** Induction of IFN-β by Mtb infection. WT BMDMs were infected with Mtb, and culture supernatant was harvested 24 h after infection and measured by IFN-β ELISA. IFN-γ priming was performed as in B. **(D)** Exogenous type I IFN exacerbates Mtb-induced cell death. BMDMs were left untreated (top panels) or primed with 10 ng/ml IFN-γ overnight before infection (bottom panels). After infection for 4 h, BMDMs were treated with indicated cytokine (IFN-α, 1,000 U/ml; IFN-β: 100, 500, and 1,000 U/ml) throughout the experiment. Cell viability was determined by measuring ATP levels on 3 (left panels) or 4 (right panels) d postinfection (d.p.i.). **(E)** Blocking type I IFN signaling partially inhibited cell death in 129S2 BMDMs. Cells were pretreated with indicated antibodies (20 µg/ml) for 2 h before infection, and antibody treatment remained throughout the experiment. Cell viability was determined by measuring ATP levels on 4 d.p.i. Data are means ± SD of three technical replicates (B–E) and representative of two independent experiments. *, P < 0.05; **, P < 0.01; ***, P < 0.001 (two-tailed unpaired Student’s *t* test). For D, statistical analysis was performed against the infected, untreated group of the same genotype. ns, not significant.

For an unbiased identification of host genes involved in the Mtb-induced death of macrophages, we performed a genome-wide CRISPR-Cas9 screen in Mtb-infected RAW264.7 cells, a mouse macrophage cell line widely used in Mtb studies. Among 19,150 mouse protein coding genes targeted by 87,897 guide RNAs (gRNAs), we identified 11 potential candidate genes whose deletion was protective (Fig. 2 A). Among these genes, only multiple components of the type I IFN signaling pathway, including *Ifnar1*, *Ifnar2*, *Jak1*, *Stat1*, *Stat2*, and possibly spleen-associated tyrosine kinase*,* are clustered together (Fig. 2 B), suggesting the importance of this pathway in mediating Mtb-induced macrophage death. Using a CRISPR-Cas9 genome editing tool, we generated *Ifnar2*^−/−^ RAW264.7 cells (Fig. S2 A) and confirmed the essentiality of *Ifnar2* for Mtb-induced death of these macrophages, without inhibiting the uptake or growth of Mtb, whether the macrophages were infected at a lower or higher MOI (Fig. 2, C–E).

### Type I IFN signaling is important for the death of Mtb-infected primary macrophages

**Figure 3.**
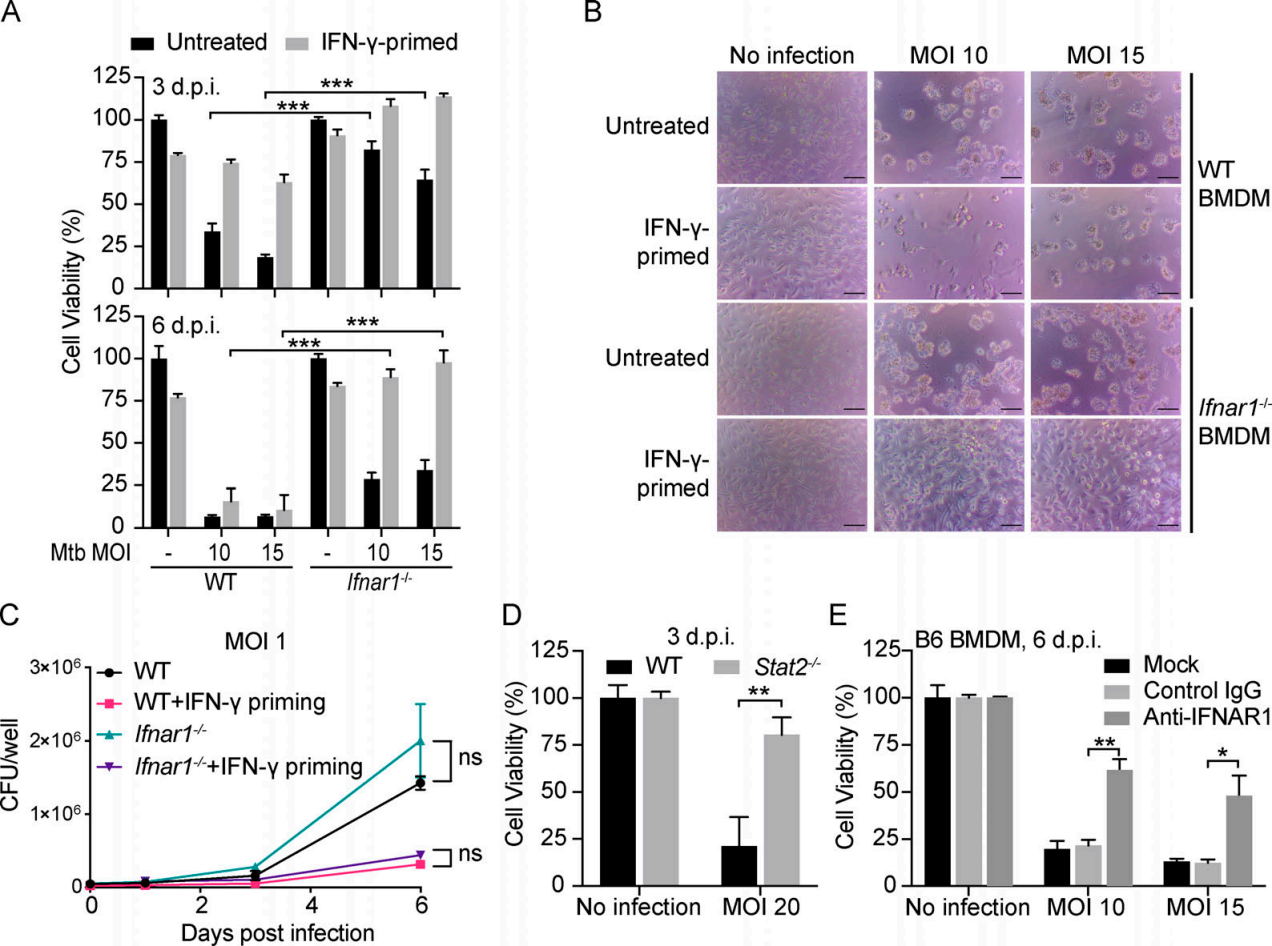
**Type I IFN signaling is important for death of Mtb-infected primary macrophages.** **(A–C)** Results for primary WT and *Ifnar1*^−/−^ BMDMs infected with Mtb at the indicated MOI. **(A and B)** Effects of *Ifnar1*^−/−^ in BMDMs on Mtb-induced cell death as assessed by intracellular ATP content on 3 (D, top) and 6 d postinfection (d.p.i.; D, bottom). **(B)** Photomicrographs of cells on day 6 post infection. Scale bars, 50 μm. **(C)** Effects of *Ifnar1* deficiency on intracellular replication of Mtb in BMDMs when infection is at an MOI of 1. IFN-γ priming at 10 ng/ml overnight. **(D)** Effects of *Stat2* deficiency on Mtb-induced BMDM death. Cells were infected with Mtb at the indicated MOI, and cell viability assays were performed on day 3 by measuring ATP levels. **(E)** Blocking type I IFN signaling partially inhibits cell death. Cells were pretreated with indicated antibodies (20 µg/ml) for 2 h before infection, and antibody treatment remained throughout the experiment. Cell viability was determined by measuring ATP levels on 6 d.p.i. Data are means ± SD of three (A and C–E) technical replicates. *, P < 0.05; **, P < 0.01; ***, P < 0.001 (two-tailed unpaired Student’s *t* test). Data are representative of two independent experiments. ns, not significant.

We then returned to the study of primary BMDMs, which provide a more physiological context than cell lines do. Death of BMDMs from *Ifnar1*^−/−^ mice was greatly delayed but not completely abolished compared with WT BMDMs, without a detectable difference in Mtb uptake or growth at either a low or high MOI (Fig. 3, A–C; and Fig. S2 B). When IFN-γ was added to activate macrophages to restrict Mtb growth (Fig. 3 C), *Ifnar1*^−/−^ cells survived dramatically better than any of the other Mtb-infected groups studied (Fig. 3, A and B). Deficiency of Stat2, which participates in type I IFN signaling, mimicked Ifnar1 deficiency in delaying the Mtb-induced death of BMDMs (Fig. 3 D).

BMDMs produced no detectable type I IFNs without infection but secreted IFN-β when infected with Mtb (Fig. S2 C), consistent with numerous reports that Mtb induces macrophages to produce type I IFNs (Cheng and Schorey, 2018; Collins et al., 2015; Dey et al., 2015; Manca et al., 2005; Stanley et al., 2007; Wassermann et al., 2015; Watson et al., 2015). Addition of exogenous IFN-β or IFN-α further enhanced the Mtb-induced cell death of WT but not *Ifnar1*^−/−^ BMDMs (Fig. S2 D). Temporarily blocking type I IFN signaling with anti-IFNAR1 monoclonal mAb partially protected WT B6 and 129S2 BMDMs from Mtb-induced death (Fig. 3 E and Fig. S2 E), confirming the importance of type I IFN signaling in this type of macrophage death.

### Blocking type I IFN signaling protects Mtb-infected mice

Given the evidence that type I IFNs may be detrimental to a host with TB, several investigators have tested the effect of genetically deleting *Ifnar1* in mouse models of TB. This has had limited impact in C57BL/6 mice (Antonelli et al., 2010; Desvignes et al., 2012; Mayer-Barber et al., 2011; Moreira-Teixeira et al., 2016; Stanley et al., 2007). However, the whole blood transcriptome signature of Mtb-infected 129S2 mice more closely resembles that of humans with TB (Domaszewska et al., 2017), and Dorhoi et al. (2014) reported that *Ifnar1* deficiency on the 129S2 background was protective. Therefore, we decided to test the impact of anti-IFNAR1 mAb or isotype-matched control IgG in 129S2 mice.

**Figure 4.**
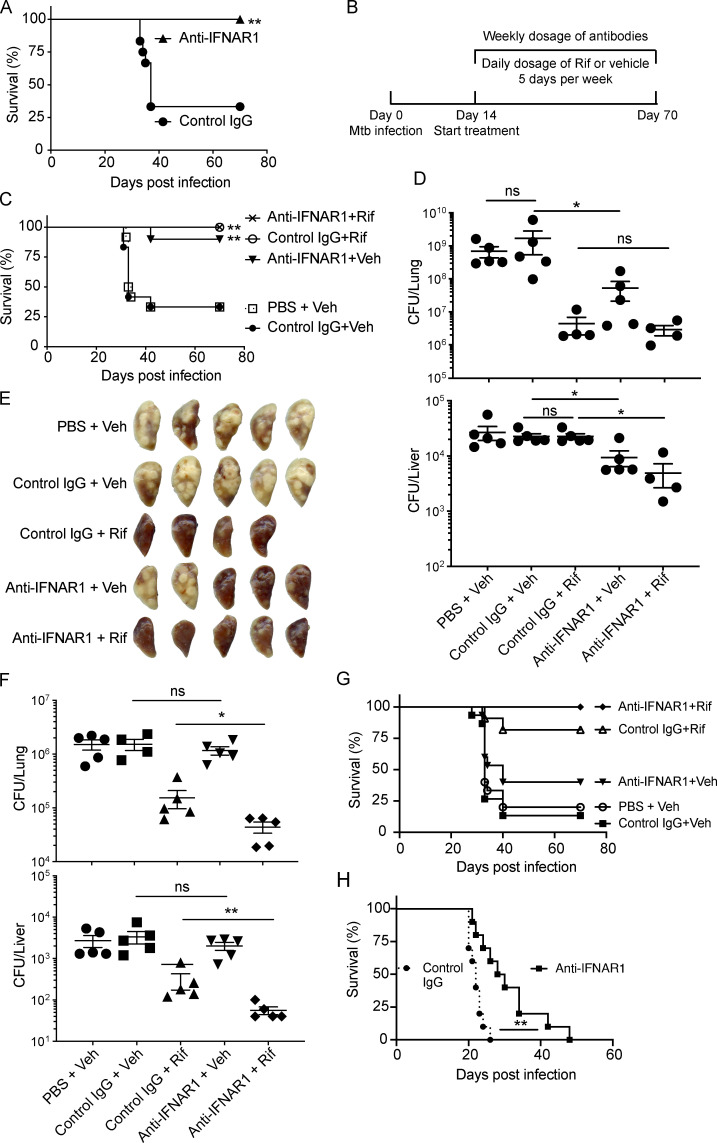
**Prophylactic or therapeutic use of anti-IFNAR1 protects mice from Mtb and augments effect of Rif.** 129S2 mice were infected on day 0 with ∼300 CFU of Mtb H37Rv for A–E, ∼180 CFU for F, ∼450 CFU for G, and ∼1,700 CFU for H. **(A)** Effects of prophylactic administration of anti-IFNAR1 on mouse survival. Mice were given 2.5 mg/mouse of isotype-matched control IgG mAb or anti-IFNAR1 mAb by intraperitoneal injection 1 d before infection followed by a weekly dose of 0.5 mg/mouse. *n* = 12 for control IgG, and *n* = 9 for anti-IFNAR1. **(B–G)** Effects of therapeutic treatment with anti-IFNAR1 mAb on mouse TB. **(B)** Scheme of the experimental setup. Mtb-infected mice started to receive PBS or antibody with (C–G) or without (H) Rif treatment on day 14. **(C)** Survival of mice (*n* = 12 for PBS + Veh and control IgG + Veh, *n* = 8 for control IgG + Rif, and *n* = 12 for anti-IFNAR1 + Veh and anti-IFNAR1 + Rif). **(D)** Mtb CFU in lungs (top) and livers (bottom) on day 33. **(E)** Gross lung pathology on day 33. **(F)** Mtb CFU in lungs (top) and livers (bottom) on day 51 when mice were infected with ∼180 CFU on day 0. **(G)** Survival of mice infected with an initial dose of ∼450 CFU. *n* = 15 for PBS + Veh, control IgG + Veh, and anti-IFNAR1 + Veh; and *n* = 10 for control IgG + Rif and anti-IFNAR1 + Rif. **(H)** Survival of mice infected with an initial dose of ∼1,700 CFU. *n* = 10 for each group. Mtb burden in mouse organs was represented as means ± SEM and analyzed by two-tailed Mann-Whitney test. Mouse survival assays were analyzed by log-rank (Mantel-Cox) test. *, P < 0.05; **, P < 0.01. ns, not significant.

**Figure S3.**
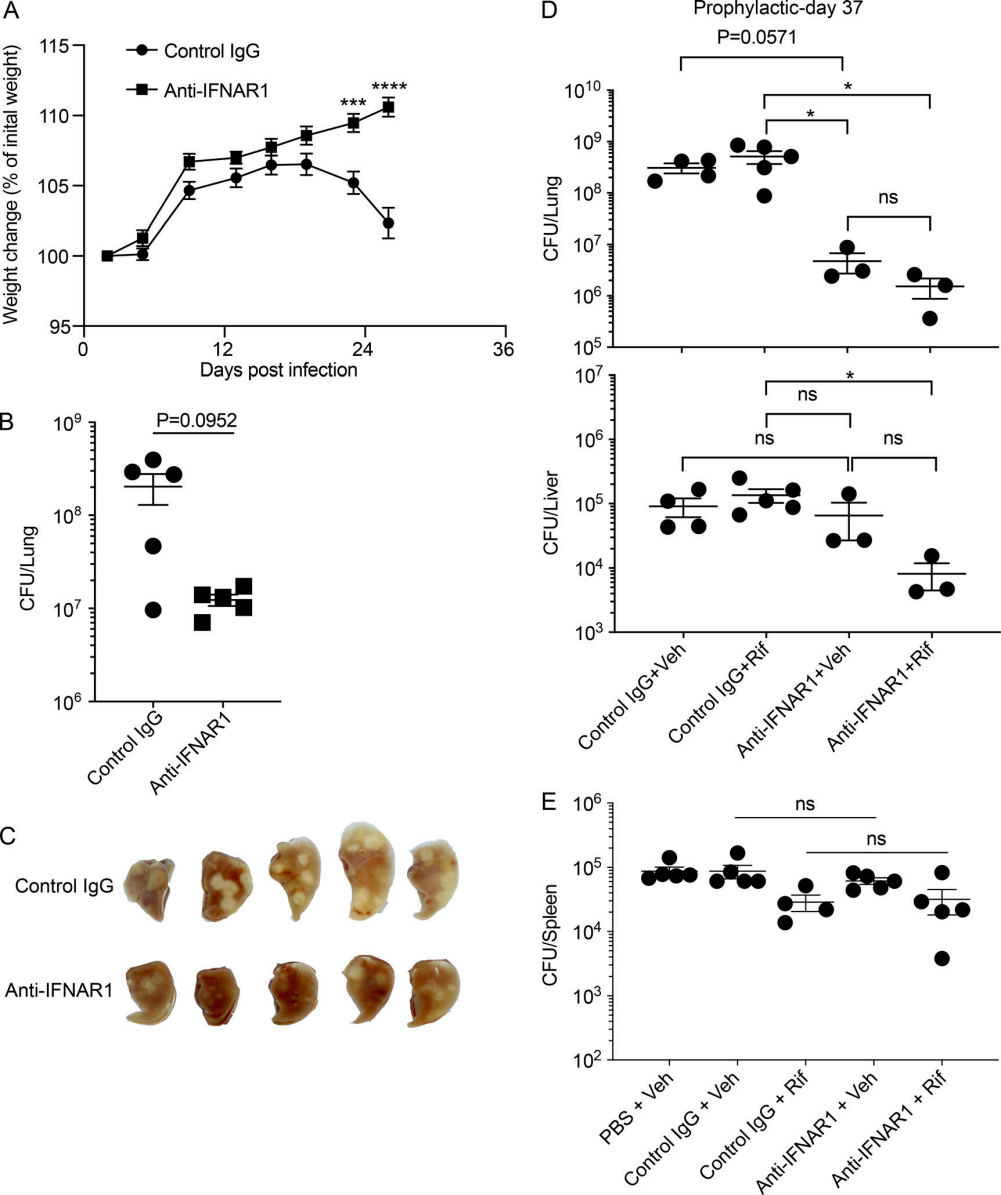
**Prophylactic and therapeutic administration of anti-IFNAR1 protects mice from TB.** Related to Fig. 4. **(A–D)** 129S2 mice were given 2.5 mg/mouse of isotype-matched control IgG mAb or anti-IFNAR1 mAb by intraperitoneal injection 1 d before infection, followed by a weekly dose of 0.5 mg/mouse. Mice were infected with ∼300 CFU of Mtb H37Rv on day 0 and started to receive Veh or 3 mg/kg Rif treatment on day 28. **(A)** Weight change of mice compared with their initial weight (*n* = 29 for each group). **(B)** Mtb burdens in the lungs on day 28. **(C)** Gross lung pathology on day 28. **(D)** Mtb CFU in lungs (top) and livers (bottom) on day 37. **(E)** 129S2 mice were infected with ∼300 CFU through the aerosol route and received antibody and antibiotic treatment beginning on day 14. Shown are spleen CFU of Mtb on day 33. Mtb buden in mouse organs and changes in mouse body weight were represented as means ± SEM and analyzed by two-tailed Mann-Whitney test. *, P < 0.05; ***, P < 0.001; ****, P < 0.0001. ns, not significant.

We began with a prophylactic regimen as proof of concept. Mice were given anti-IFNAR1 or control IgG 1 d before aerosol infection with ∼300 CFU of Mtb and weekly thereafter. Anti-IFNAR1 prevented weight loss and mortality (Fig. 4 A and Fig. S3 A) and reduced Mtb CFU and gross pathology in the lungs (Fig. S3, B and C).

Because host-directed therapy of TB would most likely be used as an adjunct to anti-mycobacterial therapy, we next tested the combination of anti-IFNAR1 mAbs and rifampin (Rif), a component of the 6-mo regimen used for drug-sensitive TB. Prophylactic and ongoing weekly administration of anti-IFNAR1 mAb was complemented by a daily dose of 3 mg/kg Rif beginning on day 28 after infection. Although Rif alone had little effect on Mtb burden by day 37 (that is, after 9 d of treatment), the combination of Rif with anti-IFNAR1 afforded a greater reduction in CFU in lungs and livers than Rif alone, suggesting the potential value of combination therapy (Fig. S3 D).

Next, we tested the effect of administering anti-IFNAR1 mAb beginning after TB disease was well established. 129S2 mice infected with ∼300 CFU of Mtb were given anti-IFNAR1 mAb, control mAb, or PBS and either Rif or its vehicle (Veh) beginning on day 14 after infection, when the lung Mtb burden had reached ∼10^6^ CFU (Fig. 4 B). About two thirds of the mice treated with PBS + Veh or control IgG + Veh succumbed to TB, while 90% of mice treated with anti-IFNAR1 + Veh survived (Fig. 4 C). In these experiments, we used Rif at 7.5 mg/kg, which sufficed to keep the mice alive for the duration of the experiment (Fig. 4 C) and lowered the Mtb burden (Fig. 4 D, top panel). On day 33, the bacterial load in the lungs of mice treated with anti-IFNAR1 + Veh was nearly 1.5 log10 lower than in those receiving PBS + Veh or control IgG + Veh (Fig. 4 D, top panel). The bacterial load reduction was consistent with the less severe pulmonary pathology in anti-IFNAR1–treated groups (Fig. 4 E). There was little change in spleen CFU (Fig. S3 E), but blocking type I IFN signaling, especially when combined with Rif treatment, yielded a statistically significant reduction in bacterial load in the liver, where Rif alone had no measurable impact (Fig. 4 D, bottom panel).

Next, we tested the impact of lower and higher bacterial burdens at the onset of infection, because the size of the inoculum impacts the rate of disease progression in mouse models of TB and can influence the results of specific interventions (e.g., Amaral et al., 2019). When mice were infected with less than ∼200 CFU and then treated as in Fig. 4 B, all of them survived for the duration of the experiment, but the combination of anti-IFNAR1 mAb and Rif yielded the lowest Mtb load in the lungs and livers (Fig. 4 F). When mice were infected with a mean inoculum of 450 CFU and treated as in Fig. 4 B, the death rates increased to 87% and 80% for control IgG + Veh and PBS + Veh treatments, respectively, and reached 18% for Rif alone. However, anti-IFNAR1 treatment alone lowered the death rate to 60%, and the combination of anti-IFNAR1 and Rif protected all the animals (Fig. 4 G). Finally, mice were infected with an extremely high bacterial inoculum (mean, 1,700 CFU). By the time treatment began on day 14, the lung Mtb load had already reached nearly 10^7^ CFU. Mice in the control groups succumbed within 30 d, while anti-IFNAR1 significantly prolonged survival (Fig. 4 H).

In sum, blocking type I IFN signaling was effective against TB when treatment started after disease was established, whether the disease was progressing slowly or rapidly, and augmented the benefit of Rif.

The mechanism of Mtb-induced macrophage cell death has been an enigma. The study of different mycobacterial species, different Mtb strains, and different macrophage sources are contributing factors. Using mouse BMDMs infected with virulent Mtb of the H37Rv strain, we confirmed that Mtb infection inhibited apoptosis (Behar et al., 2010), perhaps because it induced c-FLIP. We failed to detect activation of necroptosis triggered by Mtb infection alone, and we excluded the role of pyroptosis, parthanatos, ferroptosis, or autophagy in Mtb-induced macrophage death under the conditions studied. This suggests that the Mtb-induced macrophage cell death studied here is likely to proceed by a novel mechanism. The Mtb-infected macrophages shrink, adopt a crenellated appearance, and then disappear as recognizable cellular units in conjunction with the appearance of small particulate material in the medium, suggesting a form of lytic or necrotic death whose signaling and execution deserve further study.

We discovered that type I IFN signaling is an important mediator of Mtb-induced macrophage cell death. Type I IFN signaling has been implicated in host cell death in *Salmonella typhimurium* infection, where it promoted macrophage necroptosis in a manner dependent on RIPK1 and RIPK3 and led to poor control of the infection (Robinson et al., 2012). However, the notion that type I IFN signaling is detrimental to a host with TB has not previously been directly linked to macrophage cell death (Moreira-Teixeira et al., 2018; Trinchieri, 2010).

Type I IFNs affect many cells in diverse ways, and we do not propose that triggering the death of Mtb-infected macrophages is the most important specific mechanism of its adverse effects in TB; however, it is one of the first specific mechanisms identified. Not only does the death of infected macrophages release the pathogen to infect other cells, but the release of host cell contents may trigger tissue-damaging inflammation. Absence of *Ifnar1* in 129S2 mice markedly reduced migration of inflammatory monocytes and neutrophils to the lungs (Dorhoi et al., 2014), and prophylaxis with anti-IFNAR1 mAb protected mice subsequently infected with virulent *Mycobacterium bovis* (Wang et al., 2019). Type I IFNs impaired the activation of macrophages by type II IFN (IFN-γ; Yoshida et al., 1988), which is essential for controlling TB in mice (Cooper et al., 1993; Flynn et al., 1993) and people (Jouanguy et al., 1996; Jouanguy et al., 1997), and this was also seen in the context of infection with *Listeria monocytogenes* (Rayamajhi et al., 2010) and *Mycobacterium leprae* (Teles et al., 2013). In vitro, we found that exposure to IFN-γ led to less type I IFN production after Mtb infection (Fig. S2 D).

Our work supports a recent report that inhibition of type I IFN signaling in vivo protects mice against TB (Ji et al., 2019). In the present study, benefit was seen with onset of treatment at a higher bacterial burden and with less frequent dosing of anti-IFNAR1 mAb. The major new finding in our mouse studies was that anti-IFNAR1 mAb augmented the protection afforded by Rif, supporting the potential therapeutic value of blocking type I IFN signaling as an adjunct to antibiotic treatment. The question can now be asked: what specific sub-routines in the type I IFN signaling pathway in Mtb-infected macrophages lead to their death and by what molecular machinery? Can small-molecule inhibitors and macrophage-specific conditional knockouts thereof contribute to protection of mice from TB and shorten the duration of an otherwise optimal multidrug regimen without resorting to blocking type I IFN signaling more broadly?

The centrality of type I IFN signaling in macrophage cell death triggered by Mtb infection provides a new angle from which to study the role of type I IFNs in bacterial pathogenesis. Chemical-biological and genetic studies are underway to delve further into the mechanism. Knowledge of specific steps in this pathway may contribute to the design of host-directed therapies of bacterial infections.

## Materials and methods

### Cell lines and primary macrophages

RAW264.7 (ATCC; Cat# TIB-71, male), HEK293T (ATCC; Cat# CRL-3216, female), HT-1080 (ATCC; Cat# CCL-121, male), and L929 (ATCC; Cat# CCL-1, male) were cultured in DMEM supplemented with 10% FBS, 2 mM L-glutamine, 10 mM Hepes, pH 7.5, and 1 mM sodium pyruvate at 37°C in a 5% CO_2_ incubator. L929-conditioned medium (LCM) was filtered through a 0.22-µm sterile unit with a polyethersulfone membrane, aliquoted, and frozen at −30°C. 2 d before Mtb infection, RAW264.7 cells were preadapted to DMEM supplemented with 2% FBS, 2 mM L-glutamine, 10 mM Hepes, pH 7.5, and 1 mM sodium pyruvate and maintained in this medium for the duration of infection unless otherwise stated.

To prepare BMDMs, mouse femurs were flushed with DMEM and the cell-containing supernatant was collected after allowing debris to settle at 1 *g*. The cells were centrifuged at 193 *g* for 8 min at room temperature (hereafter, all centrifugations were performed at room temperature unless otherwise specified), and the pellet was resuspended and cultured in DMEM supplemented with 10% FBS, 2 mM L-glutamine, 10 mM Hepes, pH 7.5, 1 mM sodium pyruvate, and 20% LCM at 37°C in a 5% CO_2_ incubator for 6–7 d. On the day of harvest, adherent cells were washed once with ice-cold PBS and incubated in cold PBS + 1 mM EDTA at 4°C for 10 min. Cells were dislodged by gentle pipetting, collected by centrifugation as above, resuspended in cold PBS, washed again the same way, and then cultured in DMEM supplemented with 10% FBS, 2 mM L-glutamine, 10 mM Hepes, pH 7.5, 1 mM sodium pyruvate, and 10% LCM.

### Mice

WT C57BL/6 (#000664), WT C57BL/6N (#005304), *Casp1*^−/−^ (#032662), *Gsdmd*^−/−^ (#032410), *Stat2*^−/−^ (#023309), *Casp1*^−/−^ *Casp11*^−/−^ (#016621), and Cas9 knockin (#026179) mice (all in C57BL/6 background) were purchased from The Jackson Laboratory. *Casp11*^−/−^ mice were kindly provided by J. Blander. Cas9 knockin mice were mated with WT C57BL/6 mice, and the resulting offspring carrying one Cas9 allele (Cas9-het) were used for electroporation of gRNA amplicons. Female 129S2 mice (#476) were purchased from Charles River Laboratories, rested for 2 wk, and then infected at the age of 8 wk. All mice were housed in a specific pathogen-free facility. When infected with Mtb, mice were housed in a BSL3 vivarium. All mouse experiments were approved by and performed in accordance with requirements of the Weill Cornell Medicine Institutional Animal Care and Use Committee.

### Plasmids and reagents

LentiGuide plasmid (#52963) was purchased from Addgene. Plasmids with gRNA insertions were verified by Sanger sequencing. Mouse IFN-γ (#11276905001), TNFα (#T0157), CHX (#C4859), Rif (#R3501), Nec-1 (#N9037), Erastin (#E7781), RSL3 (#SML2234), and puromycin (#P8833) were purchased from Sigma. Mouse IFN-αA (#12100-1), mouse IFN-β (#12405-1), and Mouse IFN-β ELISA Kit, High Sensitivity (#42410-1) were from PBL Assay Science. BMN 673 (#A4153), ABT-888 (#A3002), rucaparib (#A4156), and z-VAD-FMK (#A1902) were from Apexbio. Fer-1 (#17729) was from Cayman Chemical. Smac mimetic birinapant (#501015095) was from Fisher Scientific.

For neutralization on cells, mouse IgG1 κ isotype control (#16-4714-82) was from Affymetrix, and anti-mouse IFN α/β receptor I (IFNAR1; #16-5945-85) was from Life Technologies. For use in mice, mouse monoclonal anti-human IFN-γ Rα chain (as control IgG; #G737) and mouse monoclonal anti-mouse IFNAR1 (MAR1-5A3; #I-1188) were purchased from Leinco Technologies. Antibodies for β-actin (#sc-47778) and RIPK3 (#sc-374639) were from Santa Cruz Biotechnology. Antibodies for cleaved caspase-3 (Asp175; #9664S), caspase-3 (#9662S), cleaved caspase-8 (Asp387; #9429S), caspase-8 (#4927S), phospho-RIP1 (Ser166; #31122S), MLKL (#37705S), and phospho-MLKL (Ser345; #37333S) were from Cell Signaling Technology. Phospho-RIPK3 (Thr231/Ser232; #ab222320) and ATG16L1 (#ab187671) antibodies were from Abcam. RIPK1 antibody (#610459) was from BD Biosciences. Anti-FLIP (Dave-2; #AG-20B-0005-C100) was from Adipogen Corporation.

### Bacterial culture and macrophage infection

Mtb H37Rv was used in this study. Mtb was grown in Middlebrook 7H9 medium containing 0.5% glycerol, 10% oleic acid–dextrose–catalase (BD Biosciences; #212351) supplement, and 0.02% tyloxapol at 37°C in a 5% CO_2_ incubator. For infection of macrophages, Mtb cultures reaching an OD_580_ of 0.3–0.5 were centrifuged at 3,082 *g*, and the pellet was resuspended in PBS + 0.02% tyloxapol. The suspension was enriched for single cells by centrifugation at 123 *g* for 10 min with the brake set to 0. Mtb cell number was calculated by assuming 0.1 OD_580_ = 5 × 10^7^ bacteria/ml. The required number of bacteria was centrifuged for 10 min at 3,082 *g*, resuspended in mammalian cell culture medium for infection of cells, and added to macrophages to initiate infection. 4 h after the addition of Mtb, macrophage monolayers were washed twice with PBS prewarmed to 37°C, and then fresh medium was replaced. No antibiotics were used at any time in the preparation, infection, or postinfection incubation of macrophages. For measuring extracellular bacterial burden, cell culture supernatant was removed for serial dilution and plating. For measuring intracellular bacterial burden, adherent cells in 24-well plates were washed twice with PBS and then lysed with 100 µl of 0.5% Triton X-100 in PBS at room temperature for 5 min. 400 µl PBS was then supplemented, and cellular content was mixed well by pipetting up and down. The lysates were used for serial dilution and plating on 7H11+glycerol+OADC agar plates.

### Cell viability assays

Cell viability was measured in two ways, as indicated in the figure legends. The viability of BMDMs was assayed by measuring ATP levels using a Promega CellTiter-Glo kit according to the manufacturer’s instructions. The viability of RAW264.7 cells was assayed using a Promega CytoTox 96 Non-Radioactive Cytotoxicity Assay kit in the Total Cell Number Assay mode with slight modification. The remaining adherent cells were washed once with PBS, then lysed in the kit’s 1 × lysis buffer at 37°C for 45–60 min. An aliquot of the lysate was used for the lactate dehydrogenase enzymatic assay as instructed. Percentage viability was normalized to that of uninfected cells.

### ELISA

At indicated times after Mtb infection of macrophages, cell culture supernatant was centrifuged at 21,130 *g* at 4°C for 5 min, and the supernatant was passed through a 0.22-µm cellulose acetate filter before being removed from the BSL3 facility. Neat samples were used for the IFN-β ELISA following the manufacturers’ instructions.

### CRISPR-Cas9 screen

Propagation of the Genome-wide Mouse Lentiviral CRISPR gRNA Library v1 (Addgene) followed the distributor’s guide. The lentivirus library was prepared as described (Shi et al., 2015). RAW264.7 cells (2 × 10^7^) stably expressing Cas9 protein were infected with the lentivirus library at an MOI of 0.3 with 8 µg/ml of polybrene. Cells were harvested 72 h later, reseeded at a density of 10^5^ cells/ml, and treated with 5 µg/ml puromycin to remove noninfected cells. About 8 d later, the resulting mutant cells were harvested, and 3 × 10^8^ cells were infected with Mtb at an MOI of 10 while another 10^8^ cells served as control. 4 h after Mtb infection, cells were washed twice with prewarmed PBS to remove extracellular Mtb, and fresh medium was replaced. 5 d after infection, when ∼5% of the cells remained, the medium was replaced with fresh medium (10% FBS) containing 10 µg/ml Rif and 12.5 µM moxifloxacin to kill Mtb and allow surviving host cells to recover. After another 7 d, when cells were ∼90% confluent, cells were harvested and genomic DNA was prepared as described (Shi et al., 2015). The screen was repeated three times on separate occasions.

The gRNA was amplified as in Shi et al. (2015) using primers listed in Table S1, and the quality of the amplicons was checked by a High-Sensitivity DNA Assay (Agilent) before they were sequenced in a HiSeq2500 (Illumina) using the 50-bp single-end sequencing protocol (sequencing primer listed in Table S1). The first 19 nucleotides from each sequencing read are the gRNA sequence recovered from the library. The frequency of each gRNA was calculated by dividing the gRNA read number by the total sample read number. Besides those gRNAs with 0 frequency in certain samples, the frequency of all other gRNAs ranged above 0.947. Before the fold enrichment was calculated, the frequency of each gRNA was increased by 0.01 to get rid of the “divisor being 0” error with negligible change of actual value, and then fold enrichment was calculated by comparing the processed frequency of each gRNA in the experimental sample with that in the control sample. The fold enrichment data were then analyzed by the Broad Institute CRISPR Screen Analysis Tool (https://portals.broadinstitute.org/gpp/public/analysis-tools/crispr-gene-scoring) using the Negative Binomial Distribution (STARS) method with the threshold set to a standard 10%. Because we are only interested in the biological meaning of positive selection, we used the positive selection result to calculate the average score and average −log10(P value) of three replicates. Candidate genes were determined based on the criterion that they were identified in at least two of the three independent experiments. The volcano plot of average −log10(p) versus average score was generated, and top hits were highlighted in red.

### Generation of *Ifnar2*^−/−^ RAW264.7 cells using CRISPR-Cas9

The generation of CRISPR-Cas9 knockout cell lines was adapted from a published approach (Ran et al., 2013). 2 × 10^5^ RAW264.7 cells were transfected with 0.5 µg gRNA-expressing pSpCas9(BB)-2A-eGFP plasmid, and 48–72 h after transfection, eGFP-positive cells were sorted by flow cytometry on a BD Biosciences FACS Aria II or Influx sorter and cultured till confluency. Genomic DNA was prepared from a subset of cells, and gRNA efficiency was validated by SURVEYOR assay. Cells were then sorted singly into individual wells of 96-well plates. The genotype of each clone was verified by sequencing of the PCR fragments. The gRNA sequence for *Ifnar2* is 5′-ACA​TAA​CAA​TAC​GAA​ATT​CC-3′.

### Electroporation of gRNAs into Cas9 BMDMs

LentiGuide plasmids with the correct gRNA insertion were used as templates to amplify hU6 promoter-gRNA fragments in a 50 µl × 8 PCR reaction system using NEB Phusion polymerase. The following primers were used: forward: 5′-ACC​CAG​AGA​GGG​CCT​ATT​TC-3′; reverse: 5′-CTG​TCC​CTG​TAA​TAA​ACC​CG-3′. The gRNA sequences used are negative control: 5′-GAA​CTC​GTT​AGG​CCG​TGA​AG-3′ (Sanjana et al., 2014); *Atg16l1-a*: 5′-ACC​GAA​CTG​CAC​AAG​AAG​CG-3′; and *Atg16l1-b*: 5′-GGG​TCT​GGT​TGG​CTA​CCT​CG-3′. If the intensity and purity of the PCR products were good, then the rest of the amplified DNA was purified using a Qiagen PCR purification kit and eluted with 200 µl double distilled H_2_O. 20 µl of 3 M NaAc (pH 5.2) followed by 500 µl ethanol was added to precipitate DNA at −80°C for at least 1 h. After centrifugation at 18,506 *g* at 4°C for 20 min, the DNA pellet was washed twice with 70% ethanol and allowed to air dry. DNA was then solubilized using 12 µl sterile double distilled H_2_O, and the concentration was checked using Nanodrop. The Mouse Macrophage Nucleofector Kit (Lonza) was used to electroporate 3 µg DNA into 2 × 10^7^ freshly isolated bone marrow cells from Cas9-het mice on an Amaxa Nucleofector II machine following the manufacturer’s instructions. Electroporated cells were differentiated in 20% LCM-containing DMEM as above and harvested on day 7 for use.

### Aerosol infection

Mtb H37Rv was grown in Middlebrook 7H9 medium containing 0.5% glycerol, 10% albumin–dextrose–saline supplement, and 0.05% Tween-80 at 37°C in a 5% CO_2_ incubator. Log phase cultures were centrifuged, and the pellet was resuspended in PBS + 0.05% Tween-80. A suspension enriched in single cells was prepared as above. The required volume was centrifuged for 10 min at 3,082 *g*, and the pellet was resuspended in PBS. This Mtb inoculum was used to infect mice using a Glas-Col Inhalation Exposure System.

### Administration of antibodies and antibiotic

The schedule of administration of antibodies, Rif, or Veh is illustrated in the figures. Antibodies were administrated by intraperitoneal injection. For the first dose (2.5 mg), antibodies were diluted to 6.25 mg/ml in PBS, and 400 µl was injected. For later weekly doses, 0.5 mg of antibodies in 200 µl was injected. The volume of PBS injected as a control matched the volume used to administer antibodies.

Rif was solubilized in Veh (0.5% carboxymethylcellulose and 0.25% Tween-80) by sonication. 200 µl Rif or Veh was delivered to mice by oral gavage once a day for 5 consecutive days/week.

### Mouse harvest

On day 1 after infection, two to five mice were euthanized with CO_2_, and lung homogenates in PBS were plated on 7H10 agar supplemented with 0.5% glycerol and 10% OADC. CFU were counted 3 wk later to determine the initial bacterial load. On indicated days, mice were euthanized with CO_2_, and lungs, livers, and spleens were harvested. Each organ (except the upper lobe of the left lung) was homogenized in PBS, serially diluted, and plated on 7H10 agar for determination of CFU at 3 wk. The upper lobes of the left lungs were fixed in 10% formalin.

### Statistical analyses

Results of cell viability assays, ELISAs, and bacterial growth assays in macrophages are represented as means ± SD. Mtb burden in mouse organs and changes in mouse body weight are represented as means ± SEM. Data were analyzed by two-tailed Mann-Whitney test of ranks (unpaired, nonparametric *t* test) using Prism (GraphPad Software). Mouse survival assays were analyzed by log-rank (Mantel-Cox) test. P values were noted as follows: ns, not significant; *, P < 0.05; **, P < 0.01; ***, P < 0.001; ****, P < 0.0001.

### Online supplemental material

Fig. S1 displays additional data related to Fig. 1, showing that Mtb-infected macrophages do not show markers of apoptosis, and their death is not likely to involve ferroptosis, necroptosis, parthanatos, or autophagic cell death. This figure also contains the Fer-1 authentication result. Fig. S2 displays additional data related to Fig. 2 and 3, showing that type I IFNs induced by Mtb infection contribute to Mtb-triggered macrophage death. Fig. S3 displays additional data related to Fig. 4, showing that prophylactic administration of anti-IFNAR1 mAb protects 129S2 mice against TB. Table S1 contains the oligonucleotide sequences used for the CRISPR-Cas9 screen.

## Supplementary Material

Table S1 lists oligonucleotide sequences for the CRISPR-Cas9 screen.
