# Supplementary material for: Type I interferon signaling mediates Mycobacterium tuberculosis–induced macrophage death
Source: J Exp Med. 2020 Oct 30;218(2):e20200887. doi: 10.1084/jem.20200887 (PMC7608065; doi:10.1084/jem.20200887)
Supplement: Table S1 — lists oligonucleotide sequences for the CRISPR-Cas9 screen. [file JEM_20200887_TableS1.docx]

Table S1. Oligonucleotide sequences for CRISPR-Cas9 screen

| 50bp-F | 5′-CTCTTTCCCTACACGACGCTCTTCCGATCTCTTGTGGAAAGGACGAAACA-3′ |
| --- | --- |
| 50bp-R | 5′-GTGACTGGAGTTCAGACGTGTGCTCTTCCGATCTCTAAAGCGCATGCTCCAGAC-3′ |
| Index-F | 5′-AATGATACGGCGACCACCGAGATCTACACTCTTTCCCTACACG-3′ |
| Index-R1 | 5′-CAAGCAGAAGACGGCATACGAGATCGTGATGTGACTGGAGTTC-3′ |
| Index-R2 | 5′-CAAGCAGAAGACGGCATACGAGATACATCGGTGACTGGAGTTC-3′ |
| Index-R3 | 5′-CAAGCAGAAGACGGCATACGAGATTTGACTGTGACTGGAGTTC-3′ |
| Index-R4 | 5′-CAAGCAGAAGACGGCATACGAGATGATCTGGTGACTGGAGTTC-3′ |
| Index-R5 | 5′-CAAGCAGAAGACGGCATACGAGATAAGCTAGTGACTGGAGTTC-3′ |
| Index-R6 | 5′-CAAGCAGAAGACGGCATACGAGATGTAGCCGTGACTGGAGTTC-3′ |
| Sequencing primer | 5′-TCTTCCGATCTCTTGTGGAAAGGACGAAACACCG-3′ |
